# Galactocele in the Axillary Accessory Breast Mimicking Suspicious Solid Mass on Ultrasound

**DOI:** 10.1155/2017/4807013

**Published:** 2017-04-09

**Authors:** Donya Farrokh, Ali Alamdaran, Farhad Yousefi, Bita Abbasi

**Affiliations:** Faculty of Medicine, Mashhad University of Medical Sciences, Mashhad, Iran

## Abstract

Galactoceles are the most common benign breast lesions during breastfeeding period that can mimic carcinomas. We report a galactocele with malignant appearance on ultrasound in the accessory breast. The patient was a 32-year-old lactating woman presented to our hospital for considerable swelling in the left axilla. Ultrasound examination revealed a hypoechoic mass with heterogeneous echogenicity and irregular shape and margins. Sonography-guided aspiration was performed. Aspiration of milky fluid and resolution of the axillary lump after aspiration confirmed the diagnosis of galactocele. Galactocele can present as a suspicious tumoral lesion in the axillary accessory breast and diagnostic aspiration can help in correct diagnosis of this rare lesion in the accessory breast.

## 1. Introduction

Accessory axillary breast is a congenital anomaly that is commonly bilateral and does not include areola and nipple in most cases. The diagnosis of accessory breast tissue may be delayed until the first pregnancy, when hormonal fluctuations change the breast composition [1, 2]. In the pregnancy period, symptomatic axillary breast tissue becomes painfully enlarged and a galactocele may rarely develop [2]. Galactoceles can be caused by any etiology that blocks a breast duct during lactation, but, in most cases, it is the result of a benign condition.

Galactoceles are similar to ordinary cysts, but, instead of clear fluid, they contain milk. They can mimic fibroadenoma, carcinoma, and other breast masses [3]. The mammographic and sonographic appearances of a galactocele depend upon the amount of fat in the fluid, the viscosity of the fluid, and also the amount of proteinaceous material. Aspiration of milk will generally confirm the diagnosis. Here, we report a case of galactocele in the accessory axillary breast tissue masquerading a suspicious solid mass. The presence of galactocele was confirmed after fine needle aspiration (FNA) of the axillary mass.

## 2. Case Presentation

A 32-year-old woman who was breastfeeding her first baby for 6 months, presented to our Breast Clinic with the complaint of a palpable left axillary lump. She had noticed a lump in her left axillary region before the pregnancy, which became enlarged during the pregnancy and breastfeeding periods.

**Figure 1:**
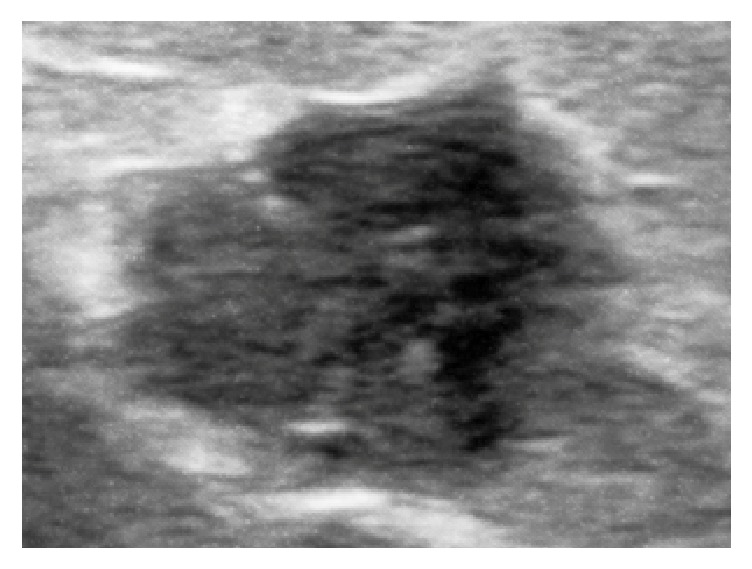
Ultrasound examination of the axillary mass reveals a hypoechoic mass with irregular margins.

Physical examination revealed a 4 cm mass in the subcutaneous tissue of the left axilla. The mass was nontender and unattached to skin. There were no evidences of inflammation such as skin color discoloration in the left axilla or fever. Ultrasound examination of the axillary region revealed a hypoechoic, well-defined mass with irregular shape and margins and nonparallel orientation measuring 2.5*∗*3.5*∗*4 cm which was suspicious for a malignant lesion (Figure 1).

Color Doppler ultrasound was performed and did not show vascular flow in the axillary mass. Ultrasound examination of the left breast did not reveal any significant abnormality and lymphadenopathy was not seen in the axilla.

There was no significant abnormality at the right breast and axilla except for the proliferative changes of glandular tissue which is a normal finding during lactation and was seen in both breasts.

**Figure 2:**
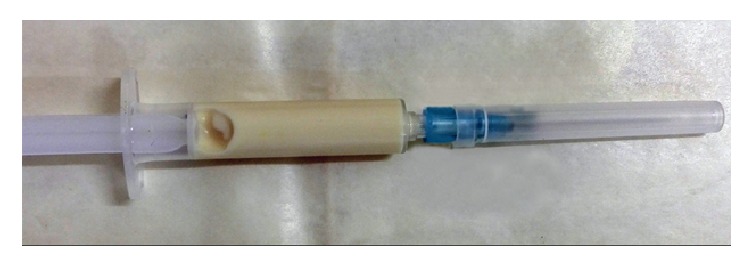
A milky fluid was aspirated with fine needle aspiration of the axillary mass.

Considering that the patient was in breastfeeding period, our first diagnosis was a galactocele in the accessory breast, but because the sonographic appearance of the axillary mass was not consistent with the ultrasound criteria of a true simple cyst or a typical benign lesion, both the clinician and the patient were concerned and insisted on performing an interventional procedure to rule out possible malignant nature of the lesion. Ultrasound-guided aspiration using a 20-gauge needle was performed for confirming an axillary galactocele. Milky fluid was aspirated and the mass disappeared completely (Figure 2).

The aspirated materials were sent for laboratory evaluation. Cytopathologic examination was negative for malignant cells. Based on these findings, the diagnosis of a galactocele of the axillary accessory breast was made.

**Figure 3:**
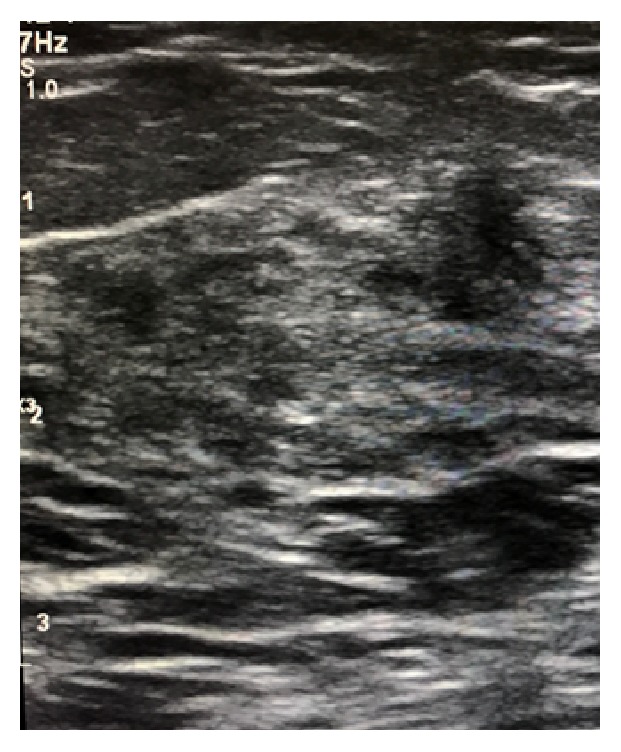
Proliferative changes in the accessory breast tissue in the axillary region. There is no evidence of mass lesion.

Additional diagnostic investigations were not indicated in our patient and she was reassured to have a follow-up sonographic examination after 3 months. She returned 4 months later and ultrasound examination was performed. There was no evidence of any left axillary mass and accessory breast tissue with proliferative changes was noted (Figure 3).

## 3. Discussion 

Galactoceles are benign lesions of the breast that represent encysted collections of milk products. They are mostly detected during lactation or in the third trimester of pregnancy. However, in rare cases, the condition may occur after breastfeeding has stopped, as milk is retained and becomes stagnant within the lactiferous ducts [1, 3, 4]. The presence of galactocele in adult males and young infants has been rarely reported [2, 4]. The presence of galactocele in the axillary accessory breast is a rare occurrence [4, 5].

Although it is most commonly located in the axilla, ectopic or accessory breast tissue may be seen anywhere along the thoracoabdominal milk line. This line extends from the axillary region down to the groin [6]. Axillary accessory breast usually presents as bilateral swellings in the axilla. Various lesions have been reported in the accessory breast in the literature including simple cyst, inflammatory lesions and mastitis, atypical hyperplasia, fibroadenoma, and rarely carcinoma [1].

Galactoceles are the most common benign breast lesions in lactating women [2, 3]. Galactoceles can mimic fibroadenoma or breast carcinoma, but they are always benign and do not increase the risk of breast cancer in any way. Galactoceles may have several causes. Three main factors are required to make a galactocele including secretory breast epithelium, present or previous prolactin stimulation and ductal obstruction. Breast surgery, oral contraceptives, and transplacental prolactin passage are reported as other possible causes in creating a galactocele [3].

Clinically, the mass is usually firm and nontender and presents as a tumoral lesion on physical examination. The patient usually notes the lesion during lactation or some period after lactation [2, 3].

The imaging appearance of galactocele depends on the amount of fat and proteinaceous material present in the cystic lesion and also the viscosity of the fluid. Pseudolipoma is the name given to the galactocele when the fat content is very high and appears as a completely radiolucent mass [7, 8]. The typical mammographic features of galactocele are a mass with fat-fluid level caused by fat and water. Fat-fluid levels are usually seen on the mediolateral mammographic view with the patient in upright position and a horizontal X-ray beam [2].

The interpretation of mammography is usually difficult in young woman particularly during breastfeeding period as mammography is usually very dense in these women. Ultrasound is the imaging method of choice to evaluate breast masses during pregnancy and lactation and mammography should be performed only in special circumstances [7, 8].

The ultrasound appearance of galactocele also depends on the amount of fat and water contents. Galactoceles with various amount of old milk, water, and proteinaceous materials may present as a heterogeneous mass with a pseudo-solid appearance, containing hypoechoic and hyperechoic materials or a complicated cyst-like lesion mimicking breast cancer, but well-defined and distinct margins would suggest a benign lesion [3, 7].

Salvador et al. reported a wavy line separating the mass into hyperechoic and hypoechoic portions or fat-fluid level [9]. Kim et al. reported that, in their series, about 4.6% of breast masses with BI-RADS category 4 on ultrasound in women during breastfeeding period were proven to be galactocele after performing core needle biopsy [3]. In rare instance when galactocele presents as a solid tumor, multiple differential diagnosis including benign fibroadenoma and invasive carcinoma should be considered [3, 10]. In general, an ultrasound-guided fine needle aspiration (FNA) and/or core needle biopsy should be performed if a lesion does not have the typical imaging appearance of galactocele or a benign breast lesion in a lactating woman [2, 11].

Milk aspiration can be performed and cyst resolution following aspiration can be a pathognomonic sign of a galactocele. Galactoceles are not serious or dangerous but may be uncomfortable. The typical treatment for a galactocele is to leave them alone. Galactoceles usually resolve spontaneously after the hormonal change associated with pregnancy and lactation is ceased. But, in patients with true discomfort, attempts may be made to drain the galactocele through FNA. Some clinicians have proposed that the diagnostic aspiration of the fluid from the cystic mass may prove to be diagnostic and therapeutic at the same time [2].

In conclusion, the presence of galactocele as a mass in accessory axillary breast tissue is a rare occurrence but that should be kept in mind in pregnant or lactating women presenting with axillary mass. Galactoceles located in the axillary accessory breast may rarely appear as a solid suspicious mass mimicking a malignant lesion. In most cases, FNA usually confirms the correct diagnosis and can be used as a diagnostic and also a therapeutic test in these patients.
